# The Relationship between Renal Function and Plasma Concentration of the Cachectic Factor Zinc-Alpha2-Glycoprotein (ZAG) in Adult Patients with Chronic Kidney Disease

**DOI:** 10.1371/journal.pone.0103475

**Published:** 2014-07-30

**Authors:** Caroline C. Pelletier, Laetitia Koppe, Pascaline M. Alix, Emilie Kalbacher, Marine L. Croze, Aoumeur Hadj-Aissa, Denis Fouque, Fitsum Guebre-Egziabher, Christophe O. Soulage

**Affiliations:** 1 Université de Lyon, F-69600, Oullins, France; 2 INSERM, U1060, CarMeN, INSA-Lyon, Univ. Lyon-1, Villeurbanne, France; 3 Hospices Civils de Lyon, Service de Néphrologie, Hôpital E Herriot, Lyon, France; 4 Hospices Civils de Lyon, Exploration Fonctionnelle Rénale et Métabolique, Hôpital E Herriot, Lyon, France; 5 Hospices Civils de Lyon, Service de Néphrologie, Centre Hospitalier Lyon-SUD, Pierre-Bénite, France; University of Florida, United States of America

## Abstract

Zinc-α2-glycoprotein (ZAG), a potent cachectic factor, is increased in patients undergoing maintenance dialysis. However, there is no data for patients before initiation of renal replacement therapy. The purpose of the present study was to assess the relationship between plasma ZAG concentration and renal function in patients with a large range of glomerular filtration rate (GFR). Plasma ZAG concentration and its relationship to GFR were investigated in 71 patients with a chronic kidney disease (CKD) stage 1 to 5, 17 chronic hemodialysis (HD), 8 peritoneal dialysis (PD) and 18 non-CKD patients. Plasma ZAG concentration was 2.3-fold higher in CKD stage 5 patients and 3-fold higher in HD and PD patients compared to non-CKD controls (P<0.01). The hemodialysis session further increased plasma ZAG concentration (+39%, P<0.01). An inverse relationship was found between ZAG levels and plasma protein (r_s_ = −0.284; P<0.01), albumin (r_s_ = −0.282, P<0.05), hemoglobin (r_s_ = −0.267, P<0.05) and HDL-cholesterol (r_s_ = −0.264, P<0.05) and a positive correlation were seen with plasma urea (r_s_ = 0.283; P<0.01). In multiple regression analyses, plasma urea and HDL-cholesterol were the only variables associated with plasma ZAG (r^2^ = 0.406, P<0.001). In CKD-5 patients, plasma accumulation of ZAG was not correlated with protein energy wasting. Further prospective studies are however needed to better elucidate the potential role of ZAG in end-stage renal disease.

## Introduction

Chronic kidney disease (CKD) and especially end-stage renal disease (ESRD) are associated with increased plasma concentration of numerous adipokines presumably as the result of a decreased glomerular filtration and/or a blunted renal degradation. Indeed, plasma concentrations of leptin [1], adiponectin [2], [3], fibroblast growth factor 21 (FGF-21) [4], retinol binding protein 4 (RBP-4) [5], chemerin [6] were found to be increased in ESRD patients. Zinc-α2-glycoprotein (thereafter referred to as ZAG) is a 43-kDa soluble glycoprotein first isolated from human plasma [7] and proposed as a tumour-derived cancer cachexia factor [8], [9]. Indeed, ZAG is overexpressed in many malignant tumours and is strongly associated with adipose tissue atrophy in cancer cachexia [10]. ZAG is however also produced by many non-malignant tissues, including white adipose tissue (WAT) and epithelia cells from liver, breast, gastrointestinal tract as well as sweat glands [11]. ZAG is described as a lipid-mobilizing factor in adipose tissue [12]. The biological activity of ZAG is linked to a cyclic AMPc-mediated signalling system through interaction with a β3/β2 adrenoreceptors [9], [10], [13], [14]. ZAG displays both lipolytic and antilipogenic effects on adipose tissue [9], [15] and stimulates uncoupling protein-1 (UCP-1) expression in brown adipose tissue, increasing thermogenesis and lipid utilization [16]. Currently, only few data are available on ZAG metabolism in kidney disease. Philipp et al [17] and Leal et al [18], [19] recently showed an increased plasma ZAG concentration in chronic hemodialysis patients, suggesting a defect of its renal clearance. However, there is no data regarding the actual plasma level of ZAG in CKD patients before initiation of renal replacement therapy. It therefore remains unclear whether the plasma accumulation of ZAG simply results from the decrease in glomerular filtration rate (GFR). The purpose of this cross-sectional study was therefore to prospectively assess the relationship between plasma ZAG concentration and GFR in patients with a large range of renal function. Plasma ZAG concentration was measured in a cohort of 71 CKD patients from stage 1 to 5, 18 non-CKD patients and correlations were explored with clinical and biochemical markers of renal function and metabolism. Plasma ZAG was further measured in 17 hemodialysis (HD) and 8 peritoneal dialysis (PD) patients.

## Materials and Methods

### Ethic statement

This research was approved by the local institutional review committee (reference D-09-17, Comité de Protection des Personnes - Recherche Biomédicale, CPP Lyon Sud-Est IV) and conducted in accordance with its ethical standards and the principles of the Declaration of Helsinki. All subjects involved in the research signed written informed consent forms prior to enrolment.

### Subjects

From November 2010 to March 2013, a total of 89 subjects (43 men and 46 women), among which 18 non-CKD subjects, were recruited from Nephrology department and Unité de Jour d’Exploration Rénale et Néphrologie (UJERN) (E. Herriot University hospital Lyon, France). Glomerular filtration rate (eGFR) was estimated using the CKD EPI formula [20], [21] and CKD stages were determined according to K/DOQI guidelines [22]. Seventeen chronic HD and 8 PD patients on renal replacement therapy for more than 6 months were further recruited at the Nephrology/Hemodialysis unit (E. Herriot University hospital, Lyon, France). Patients with severe conditions including systemic inflammation or known malignant active diseases were excluded from the study. People were classified into 8 groups: Healthy volunteers, CKD stage 1, CKD stage 2, CKD stage 3, CKD stage 4, CKD stage 5 - no renal replacement therapy, CKD stage 5 - hemodialysis, CKD stage 5 - peritoneal dialysis.

### Anthropometric and clinical measurements

Body weight was measured in light clothing without shoes to the nearest 100 g on a digital scale, corresponding to dry weights for HD patients. Height was measured in standard position with a portable stadiometer. Body mass index (BMI) was calculated as body weight divided by squared height. Body fat mass was calculated according to Deurenberg et al (1991) using the following formula: Body fat (%) = (1.20×BMI)+(0.23×Age)−(10.8×Gender) where male gender = 1, female gender = 0 [23]. CKD patients were considered having PEW if they presented at least 3 out of 4 of the following criterions, BMI<23 kg/m^2^, Albumin<38 g/L (bromcresol green assay), Pre-albumin <300 mg/L or Protein intake<0.6 g/kg.24 h according to Fouque et al (2008) [24].

### Blood sampling

After an overnight fast, blood samples were obtained by venipuncture, except for HD blood samples that were obtained immediately before and after dialysis from arterial line of the mechanical bloodstream. Blood samples were centrifuged at 3500×g for 10 min to isolate plasma supernatant which was snap frozen in liquid nitrogen and stored at −20°C until use.

### Glomerular filtration rate measurements (mGFR)

In a subset of 42 CKD stage 1 to 4 subjects, GFR was measured by the gold standard method, i.e. the urinary inulin clearance (ml/min per 1.73 m^2^), as described previously [25]. Briefly, inulin (Polyfructosan[inutest], Laevosan, Linz, Austria) was infused continuously for 4 hours after a priming dose. Blood and urine were collected every 30 minutes by peripheral venous catheter and spontaneous voiding, respectively. Inulin was measured by the enzymatic method [26]. GFR was calculated with the UV/P formula with U = urine inulin concentration, V = urinary volume and P = plasma inulin concentration.

### ZAG immunoassay

Plasma ZAG concentrations were determined with a commercially available ZAG Enzyme Immunoassay (RayBiotech Inc, Tebu-Bio, Le-Perray-en-Yvelines, France) according to the manufacturer's instructions. The detection limit was 21 pg/ml and intra and inter-assay coefficient was 3.6% and 3.2% respectively. Measurements were performed at least in duplicate. Post-dialysis ZAG concentrations were corrected for hemoconcentration using plasma protein concentrations assayed according to the method of Bradford [27] with bovine serum albumin as standard. The correction was performed using the following equation: [ZAG]_Corrected_ = ([Proteins]_pre-HD_/[Proteins]_post-HD_)×[ZAG]_post-HD_ with [Proteins]_pre-HD_, plasma protein concentration before haemodialysis session, [Proteins]_post-HD_ and [ZAG]_post-HD_, plasma protein and plasma ZAG concentrations after haemodialysis session, respectively.

### Other biochemical measurements

Plasma urea, bicarbonate, hemoglobin, protein, glucose, HDL and LDL cholesterol, triglycerides, and C-reactive protein (CRP) were measured by standard laboratory methods in a certified laboratory. Albumin was measured by immunonephelometry. Creatinine assay was performed by enzymatic method (Roche, Meylan, France), with calibrators assigned by an isotope-dilution mass spectrometry. Plasma glycerol concentration (an index of white adipose tissue lipolysis) was measured using a colorimetric enzymatic assay (Glycerol assay, R-Biopharm, Saint-Didier, France) and normalized to body fat mass as described by Axelsson et al (2011) [28].

### Statistical analysis

Data were analysed using Graphpad Prism 5.0 (GraphPad softwares, La Jolla, USA) and Statview 4.5 (Abacus concept, Berkeley, USA) softwares. The data are expressed as mean±1 standard deviation (SD) or as median (interquartile range [IQR]) when variable were not normally distributed. Distributions were tested for normality using d’Agostino-Pearson test. Differences between groups were assessed by Kruskall & Wallis test followed when appropriate by Dunn tests. Simple comparisons were made using Mann & Whitney U test. Sex ratio and medications between groups were compared using Chi-square test. Univariate analysis was performed using the Spearman rank correlation method. A multivariate linear regression analysis was used to define the variables most predictive of circulating ZAG concentration after selection of the measures found to be associated with ZAG by univariate analysis (i.e. urea, plasma proteins, hemoglobin and HDL-cholesterol). A P<0.05 was considered as statistically significant in all analysis.

## Results

### Patient data

We recruited 71 patients with CKD from stage 1 to end-stage 5 (aged 57±17 years), 18 non-CKD patients (aged 48±19 years), 17 HD patients (aged 66±17 years) and 8 PD patients (aged 58±14 years). Their renal diseases were glomerulonephritis (N = 32 including 19 diabetes), renovascular disease (N = 22, including 14 nephroangiosclerosis), 9 tubulointerstitial disease, 5 cystic kidney diseases and of other or unknown aetiologies in 28 patients. Non-CKD patients were referred of renal function evaluation for urinary lithiasis exploration or before kidney donation. Out of the 96 CKD, HD and PD patients, 54 were on antihypertensive drugs (56.8%), 45 on lipid lowering drugs (47.3%), 32 were on beta-blockers (33.3%) and 30 on antidiabetic drugs (31.5%, amongst which 24 used insulin and 6 oral antidiabetics). The main clinical, anthropometric and biochemical characteristics of the cohort are summarized in **Table 1** and dialysis adequacy is described in **Table 2**. As expected, eGFR (estimated by CKD EPI, P<0.001), plasma creatinine (P<0.001) and urea (P<0.001) were different between groups.

**Table 1:** Baseline characteristics of non CKD, CKD, hemodialysis and peritoneal dialysis patients involved in the study (N = 114).

	Non CKD	CKD stage					HD	PD	P-value
		1	2	3	4	5			
Sex, male/female	9/9	2/6	8/12	11/7	7/5	6/7	8/9	4/4	P = 0.777
Age, y	52 (28–63)	31 (20–48)	59(48–70)	69 (58–76)	67 (54–76)	61 (58–66)	66 (53–86)	58 (46–71)	P = 0.001
Weight, kg	67.0 (53.0–73.8)	59.5 (48.2–74.5)	65.0 (58.5–76.3)	75.0 (64.5–92.3)	74.0 (64.0–85.8)	71.0 (60.0–87.5)	65.0 (55.0–77.8)	63.9 (55.5–76.8)	P = 0.156
BMI, kg/m^2^	23.4 (21.2–25.6)	20.8 (18.5–25.6)	25.3 (22.1–27.2)	26.7 (23.4–32.9)	26.2 (24.3–31.8)	24.0 (22.9–30.8)	25.8 (21.9–30.3)	23.9 (20.7–29.5)	P = 0.100
Creatinine, µM	58.5 (50.8–62.3)	53.5 (42.0–72.0)	82.5 (72.5–93.5)	137 (122–171)	301 (198–368)	456 (382–639)	643 (450–815)	641 (498–846)	P<0.001
eGFR, ml/min.1.73 m^2^	109 (99–138)	124 (94–134)	75 (68–88)	43 (33–45)	16 (14–24)	9 (8–12)	N/A	N/A	P<0.001
Urea, mM	4.7 (3.9–5.7)	4.2 (3.6–6.8)	6.1 (5.0–7.7)	13.6 (9.5–15.3)	20.7 (14.3–24.5)	24.0 (17.2–28.5)	21.4 (18.3–28.2)	16.6 (14.7–21.2)	P<0.001
Bicarbonates, mM	25.0 (23.2–27.1)	25.9 (23.9–30.5)	26.8 (24.5–28.3)	23.3 (22.1–25.8)	21.9 (18.6–25.8)	24.8 (21.8–26.8)	24.1 (22.5–25.0)	26.9 (24.4–28.7)	P = 0.002
Proteins, g/L	70.0 (65.8–76.5)	70.5 (54.3–75.8)	70.0 (66.5–75.8)	73.0 (70.3–79.3)	73.5 (70.3–75.0)	72.0 (65.0–76.0)	68.0 (66.0–72.0)	74.5 (65.5–78.0)	P = 0.385
Albumin, g/L	41.5 (37.0–42.9)	37.0 (17.0–41.0)	41.8 (37.0–43.7)	36.9 (35.0–42.5)	38.0 (33.0–40.0)	32.0 (26.5–34.5)	38.0 (33.5–40.5)	35.0 (27.0–36.0)	P = 0.009
Hemoglobin, g/L	127 (117–144)	126 (107–140)	130 (122–138)	119 (102–134)	112 (101–122)	118 (102–122)	116 (102–122)	125 (104–136)	P = 0.003
Glucose, mM	4.8 (4.3–5.4)	4.8 (4.2–5.4)	5.0 (4.5–5.7)	5.9 (4.4–7.0)	5.3 (4.8–6.3)	5.6 (4.8–6.9)	5.4 (4.4–5.8)	4.9 (4.7–8.1)	P = 0.129
Triacylglycerols, mM	0.76 (0.55–1.33)	1.74 (1.44–3.02)	1.30 (1.00–1.97)	1.46 (1.08–2.21)	1.37 (1.21–2.60)	1.61 (1.33–2.16)	1.87 (1.04–2.38)	1.32 (0.71–2.84)	P = 0.038
HDL cholesterol, mM	1.10 (0.95–1.32)	1.10 (0.91–1.57)	1.29 (0.98–1.86)	1.19 (1.00–1.36)	1.14 (0.90–1.24)	1.01 (0.87–1.1)	1.18 (0.99–1.56)	1.48 (0.69–4.03)	P = 0.449
LDL cholesterol, mM	2.38 (1.87–3.22)	2.25 (1.73–5.02)	2.83 (2.20–3.84)	1.61 (1.33–2.01)	1.44 (1.26–2.79)	2.18 (1.64–2.67)	2.02 (1.42–3.68)	2.18 (1.68–2.52)	P = 0.044
CRP, mg/L	2.9 (2.9–3.2)	3.0(2.9–7.2)	2.9 (2.9–3.1)	4.5 (2.9–7.0)	2.9 (2.9–9.6)	3.2 (2.9–11.3)	4.4 (2.9–9.2)	2.9 (2.9–2.9)	P = 0.067
Anti diabetic therapy,%	0.0	12.5	5.0	50.0	77.7	23.1	17.6	50.0	P<0.001
Lipid-lowering therapy,%	5.9	12.5	30.0	66.7	80.0	38.5	47.0	50.0	P = 0.001
RAA inhibitors,%	11.8	37.5	25.0	88.9	60.0	77.0	41.2	62.5	P<0.001
Beta blockers, %	0	0	22.2	47.1	27.3	30.8	58.8	37.5	P = 0.005

*Continuous data are expressed as median (interquartile range) and compared using Kruskall & Wallis test. Categorical data are expressed as the frequency (%) and compared using Chi square test. Abbreviations: CKD, chronic kidney disease, BMI, body mass index, eGFR estimated glomerular filtration rate, HD, hemodialysis, N/A, not applicable, PD, peritoneal dialysis, RAA, renin angiotensin aldosterone. eGFR was calculated using CKD EPI formula. Differences were considered significant at P<0.05.*

**Table 2:** Dialysis adequacy for HD and DP patients.

	Hemodialysis	Peritoneal Dialysis
Dialysis modality	9 HD/8 HDF	5 CAPD/3 CCPD
Catheter/Fistula/not documented, n =	3/12/2	NA
Time of dialysis (h/week)	12.27±0.61	NA
Blood flow (ml/mn)	340.91±24.27	NA
Kt/V	1.67±0.31	NA
Weekly Kt/V urea	NA	2.43±0.51
Weekly peritoneal clearance (L/week/1.73 m^2^)	NA	89.18±22.53
Residual renal fonction (L/week/1.73 m^2^)	NA	48.3±25.01

*Data are expressed as means ± one standard deviation. Abbreviations: CAPD, continuous ambulatory peritoneal dialysis, CCPD continuous cyclic peritoneal dialysis, HD, hemodialysis; HDF, hemodiafiltration, NA, not applicable.*

### Plasma ZAG concentration increases in ESRD patients

**Figure 1:**
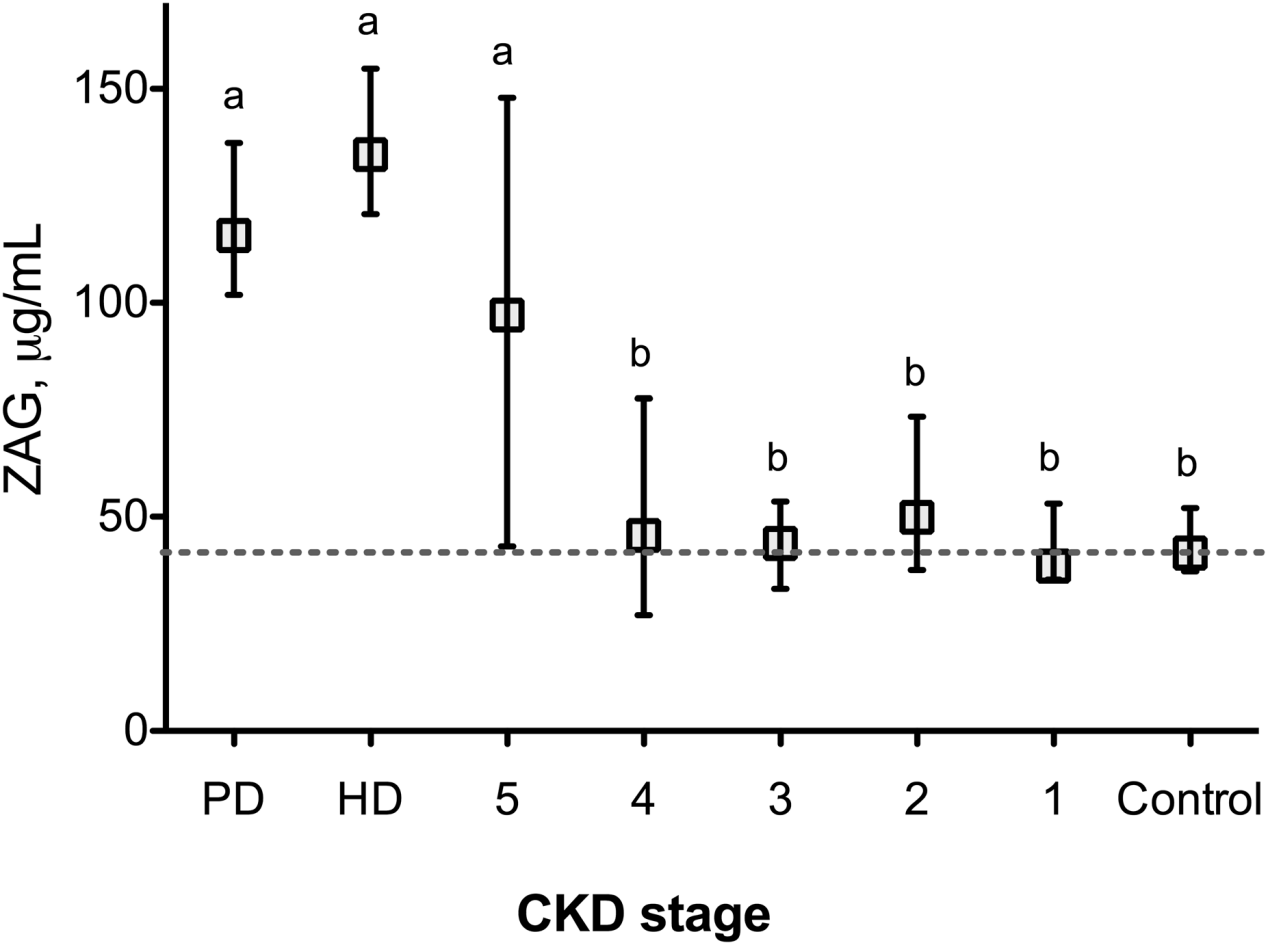
Plasma ZAG concentration is increased in end stage renal disease patients. ZAG concentration was quantified in plasma from non-CKD subjects (N = 18), chronic kidney disease (CKD stage 1 to 5, N = 71), hemodialysis (HD, N = 17) and peritoneal dialysis (PD, N = 8) patients by enzyme immunoassay. eGFR was estimated using CKD EPI formula as described in methods. Data are presented as median (interquartile range). Different letters indicate a significant difference at the P<0.05 level.

Surprisingly, plasma ZAG concentration did not gradually increase from CKD stage 1 to CKD stage 4 when compared to non-CKD patients (**Figure 1**). Plasma ZAG concentration was however 2.3-fold higher in CKD 5 (ESRD) patients as compared with non-CKD patients or CKD stage 1 to 4 patients (Kruskall & Wallis test, P<0.005). The plasma ZAG concentration was 41.5 (interquartile range (IQR), 37.3–52.0) µg/ml for non-CKD patients and 97.2 (43.1–147.9) µg/ml for CKD 5 subjects (P<0.01). No significant difference in ZAG concentration could be demonstrated depending on gender (male vs female: 56 (33–126) vs 40 (31–100) µg/ml, P = 0.096) or diabetic status (non diabetes mellitus *vs* diabetes mellitus, 46 (33–115) vs 48 (27–110) µg/ml, P = 0.908). Plasma ZAG concentration was not significantly different from non-dialyzed CKD 5 patients neither in HD patients (134.6 (120.7–154.7) µg/ml) nor in PD patients (134.6 (98.2–137.3) µg/ml). To get further insight, GFR was measured in a subset of patients (CKD 1 to CKD 5, N = 42) by the gold standard method, i.e. the urinary inulin clearance. In good agreement, no correlation was found between GFR assessed by inulin clearance and plasma ZAG concentration in patients with CKD stage 1 to 4 (Spearman r_s_ = 0.174, P = 0.283).

**Figure 2:**
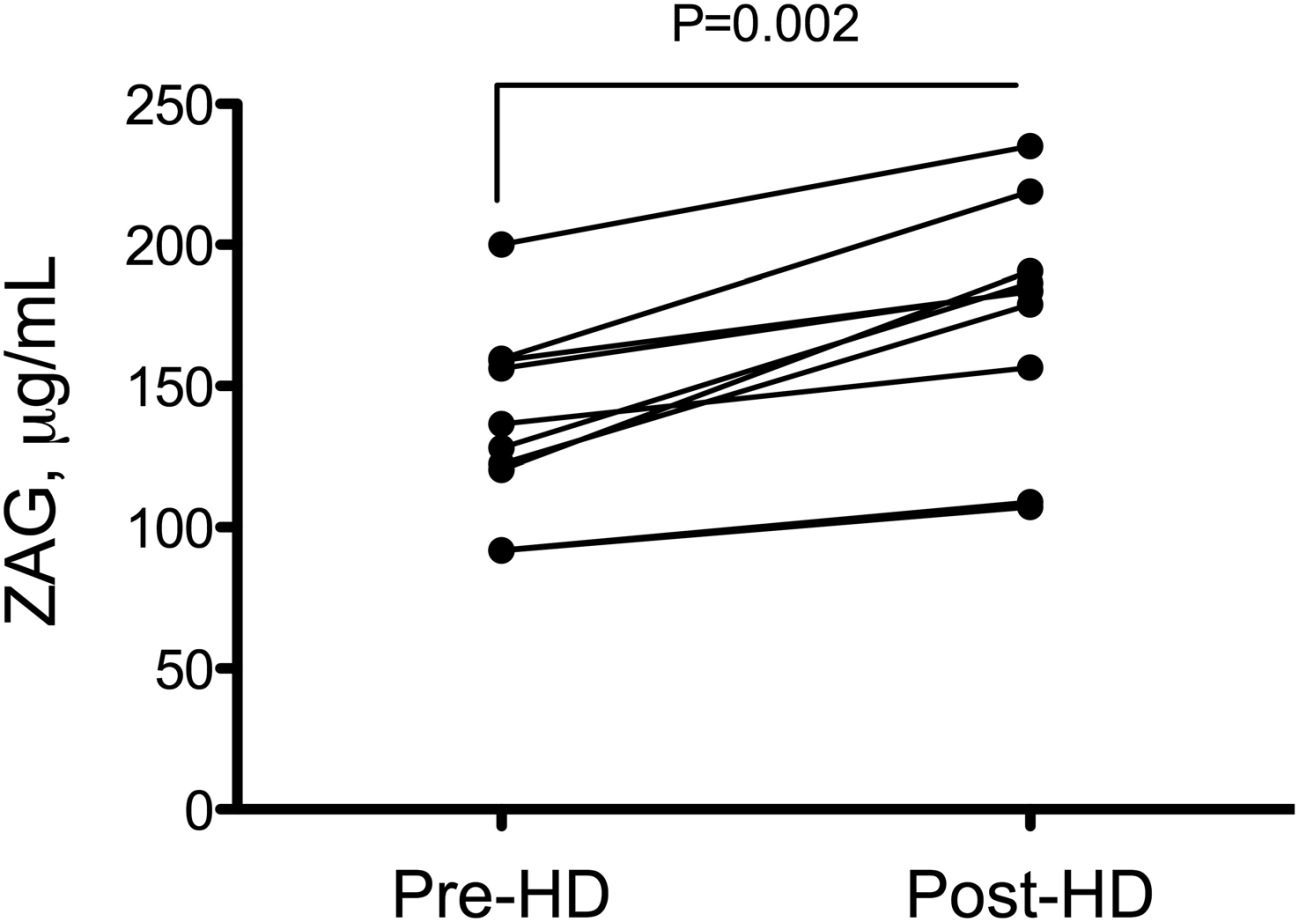
Plasma ZAG concentration increases during hemodialysis session. ZAG concentration was quantified by enzyme immunoassay before and after an hemodialysis session in ESRD patients (N = 8). ZAG concentrations were corrected for hemoconcentration as described in methods. Differences between pre and post dialysis concentrations were considered significant at the P<0.05 level (Wilcoxon test for paired samples). Abbreviation: HD, hemodialysis.

HD patients exhibited a further increase in plasma ZAG concentration after the dialysis session. The mean plasma ZAG concentration was 132.3 (113.3–159.2) µg/ml before and 196.1 (168.8–217.9) µg/ml after haemodialysis session (+48%, P = 0.002). Hemodialysis resulted in a significant hemoconcentration evidenced by the increased plasma protein concentration (68.3±6.1 vs 74.8±7.8 g/L, before and after HD, respectively, +9%, P = 0.015). However, the post-dialysis increase in ZAG concentration did not only result from hemoconcentration since normalization of ZAG concentration to plasma protein concentration did not abolish this difference (**Figure 2**). Corrected ZAG concentration after haemodialysis session was 183.7 (144.7–197.7) µg/ml (+39%, P = 0.008).

### Relationships between plasma ZAG Concentration and clinical or biochemical parameters

**Figure 3:**
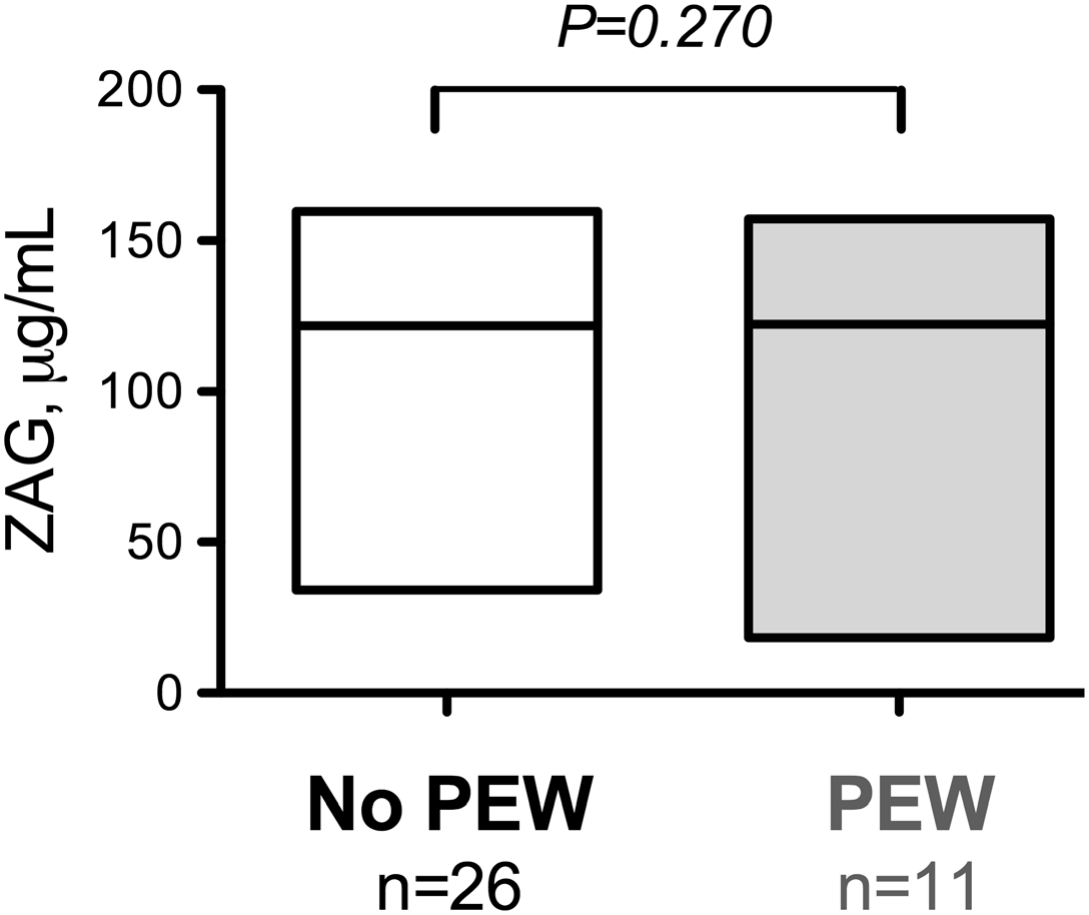
Plasma ZAG concentration in CKD-5 patients with a diagnostic of protein-energy wasting. The boxes indicate the range (i.e. min to max) and the line indicate the median. Note that no difference was found to be significant at the P<0.05 level. Abbreviation: PEW, protein-energy wasting.

**Figure 4:**
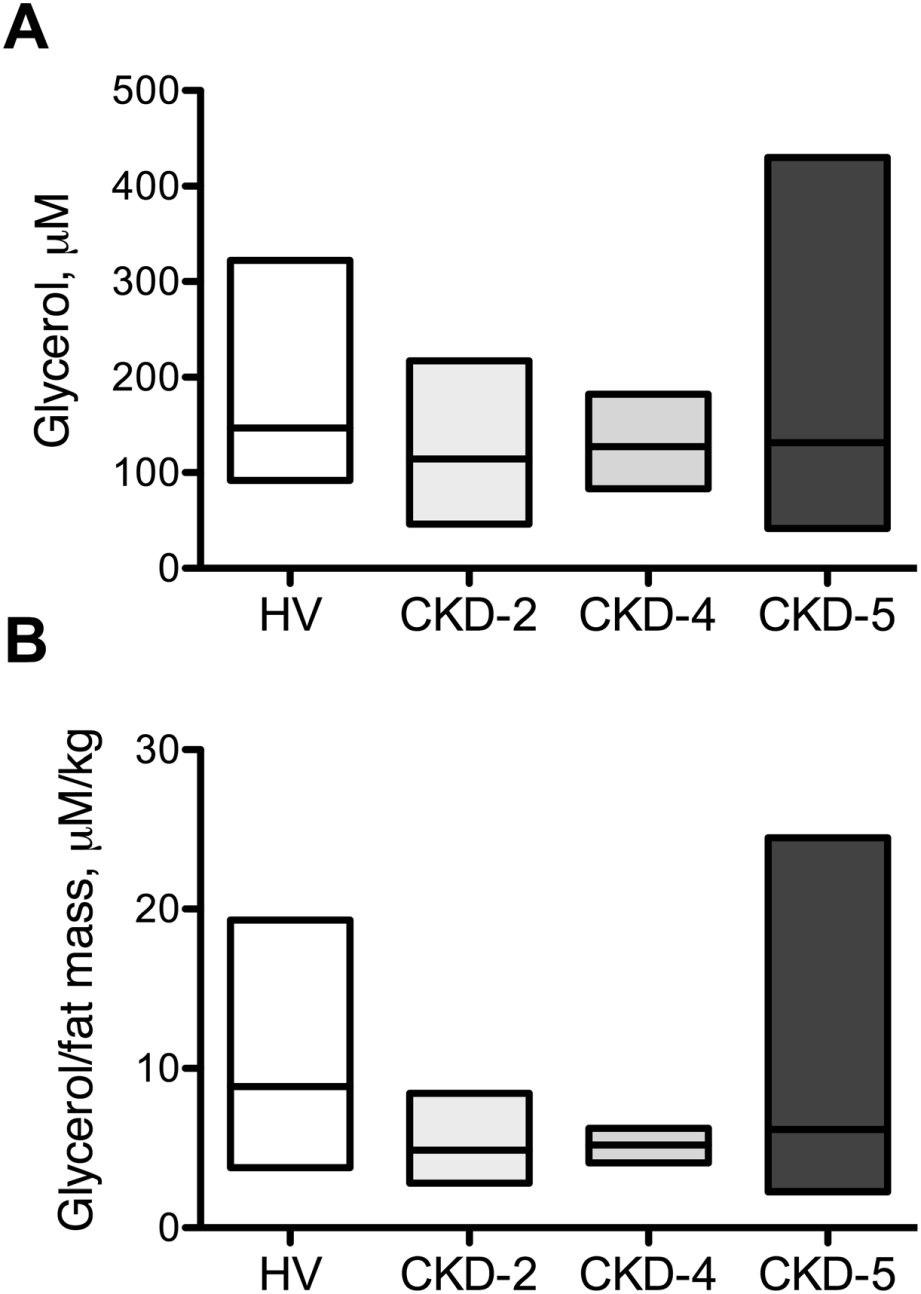
Plasma glycerol concentration in non-CKD and CKD patients. Plasma glycerol was measured as an index of lipolysis in 17 non-CKD patients, 6 CKD-2, 4 CKD-4 and 12 CKD-5 patients. **A)** plasma glycerol concentration **B)** Plasma glycerol, concentration normalized to body fat mass as described in methods. No difference was found to be significant at the P<0.05 level using Kruskall & Wallis test.

The results of the univariate correlation analysis are summarized in **Table 3**. In CKD patients stage 1 to 5, plasma ZAG concentrations negatively correlated with plasma protein concentration (r_s_ = −0.284, P<0.01), albumin (r_s_ = −0.282, P<0.05) hemoglobin (r_s_ = −0.264, P<0.05), urea (r_s_ = 0.283, P<0.01) and HDL-cholesterol (r_s_ = −0.265, P<0.05). Multiple regression analysis (**Table 4**) including urea, protein, haemoglobin and HDL-cholesterol indicated that only urea (P<0.005) and HDL-cholesterol (P<0.05) were associated with ZAG plasma concentration. Urea, proteins, haemoglobin and HDL-cholesterol explained 41% of the variance of plasma ZAG concentration (P<0.001). Out of the 38 patients CKD-5, HD or PD, 11 (29%) presented PEW according to the criterions described in the method section (see patient characteristics in **Table 5**). We therefore re-analyzed the plasma ZAG concentrations of CKD-5, HD and PD patients presenting or not PEW. No significant difference in plasma ZAG concentration was noticed between these 2 sub-groups (**Figure 3**) suggesting that ZAG accumulates in CKD but does not have any relation with PEW. Since ZAG was described to exert potent lipolytic activity, plasma glycerol was measured in a subset of 39 patients (17 non CKD and 22 CKD) as an index of lipolysis. We did not observe any difference in plasma glycerol concentration between HV and CKD patients (**Figure 4**). Additionally, no significant correlation was found between ZAG and plasma glycerol concentration.

**Table 3:** Univariate correlations with ZAG concentrations.

	rho	P-value	
Age, y	0.103	0.340	
BMI, kg.m^−2^	0.112	0.296	
CRP, mg L ^−1^	0.088	0.507	
Protein, g.L ^−1^	−0.284	0.008	**
Albumin, g.L ^−1^	−0.282	0.016	*
Hemoglobin, g.L ^−1^	−0.264	0.015	*
Bicarbonate, mM	−0.001	0.905	
Urea, mM	0.283	0.008	**
Creatinine, µM	0.274	<0.01	**
Proteinuria, g.24 h^−1^	0.257	0.061	
Glucose, mM	0.138	0.200	
HDL-cholesterol, mM	−0.265	0.034	*
LDL-cholesterol, mM	−0.097	0.448	
Triacylglycerols, mM	0.119	0.340	

*Abbreviations: BMI: Body mass index, CRP: C-reactive protein, eGFR: estimated glomerular filtration rate. Correlations were significant at P<0.05 level.*

**Table 4:** Multiple linear regression showing association with plasma ZAG concentration (r^2^ = 0.406, P<0.001).

Dependent variable: plasma ZAG				
Independent variable	β coefficient	Standard error	P-value	
Urea	2.024	0.583	<0.005	***
Proteins	−1.346	0.571	0.023	*
Hemoglobin	−0.195	0.264	0.465	
HDL-cholesterol	−27.217	11.739	0.025	*
Intercept	181.182	54.338		

*Correlations were significant at P<0.05 level.*

**Table 5:** Characteristics of CKD-5 patients with PEW.

	No PEW	PEW	
Sex, male/female	14/12	6/5	P = 0.969
Age, y	63 (54–73)	62 (47–84)	P = 0.947
Weight, kg	68.0 (57–78)	55 (51–77)	P = 0.122
BMI, kg/m^2^	27.4 (23.0–30.0)	21.8 (19.2–24.0)	P = 0.021*
Creatinine, µM	589.5 (443.3–822.5)	613.0 (407.0–683.0)	P = 0.921
Urea, mM	21.0 (17.0–27.8)	18.5 (17.1–26.9)	P = 0.642
Bicarbonates, mM	24.5 (22.8–26.0)	24.0 (22.0–25.0)	P = 0.393
Proteins, g/L	69.5 (66.8–76.3)	64.0 (59–77.0)	P = 0.079
Albumin, g/L	36.0 (33.5–39.2)	27.0 (26.0–33.1)	P = 0.001***
Prealbumin, mg/L	0.31 (0.28–0.39)	0.22 (0.18–0.32)	P = 0.013*
Hemoglobin, g/L	116.5 (103.5–123.3)	116.0 (103.0–121.0)	P = 0.907
Glucose, mM	5.5 (4.8–6.6)	5.4 (4.5–6.0)	P = 0.505
Triacylglycerols, mM	1.75 (1.29–1.56)	1.45 (1.01–1.39)	P = 0.290
HDL cholesterol, mM	1.13 (0.86–1.32)	1.04 (0.98–1.57)	P = 0.816
LDL cholesterol, mM	2.15 (1.69–3.59)	1.76 (1.32–2.56)	P = 0.113
CRP, mg/L	3.2 (2.9–9.1)	2.9 (2.9–12.0)	P = 0.912
Anti diabetic therapy, %	30.8	18.2	P = 0.668
Lipid-lowering therapy, %	45.5	46.2	P = 1.000
RAA, %	45.4	57.7	P = 0.860
Beta blocking agents, %	45.4	42.3	P = 1.000

*Continuous data are expressed as median (interquartile range) and compared using Mann & Whitney U test. Categorical data are expressed as the frequency (%) and compared using Chi square test. Abbreviations: CKD, chronic kidney disease, CRP, C-reactive protein, BMI, body mass index, PEW, protein-energy wastng, RAA, renin angiotensin aldosterone.*

## Discussion

Zinc alpha2-glycoprotein is a soluble glycoprotein acting as a cachexia factor through its activity on adipose tissue. Few studies [17]–[18], [19] recently demonstrated that plasma ZAG concentration was increased in chronic hemodialysis patients, suggesting a defect of its renal clearance. However, there is no data reporting the actual concentration of ZAG in CKD patients before initiation of renal replacement. We report in the present study that plasma ZAG concentration sharply increases in CKD 5 patients, as in patients with renal replacement therapy, but is poorly correlated to the decrease in GFR.

The CKD stage 5 patients included in the present investigation exhibited a marked elevation of plasma ZAG concentration (2.3-fold, p<0.005) that was also observed for PD patients, pre- and post-dialysis HD patients. ZAG accumulation in CKD 5 can result from a decrease of its renal clearance, an increased secretion rate by epithelia cells or both. In a pioneering work, Eckman et al suggested that the kidney could play a key role in the catabolism of ZAG [29]. Because of its rather small molecular weight (43 kDa) and size (Stokes radius 3.24 nm), a fraction of plasma ZAG normally passes through the glomerular membrane after which it is totally reabsorbed at the tubular level. They reported that impairment of renal function, with either decreased GFR or decreased tubular reabsorption, results in a rise in ZAG/creatinine clearance ratio [29]. In the present study, we did not find a strong correlation between plasma ZAG and GFR since ZAG concentration only rises in CKD stage 5. It is well known that glomerular filtration rate estimated by the CKD EPI formula may not accurately reflect renal function [30]. We therefore confirmed these findings with the direct measurement of GFR using the gold standard method of inulin clearance that yielded similar results.

To the best of our knowledge there is only scarce data in the literature regarding the fate of ZAG in the body. The actual turnover rate of ZAG in plasma is to date unknown. Many works documented the increase in plasma concentration of cytokines and hormones such as adiponectin and leptin in ESRD subjects [1]–[3], [31]. Indeed, leptin is cleared from the bloodstream by the kidney through glomerular filtration followed by metabolic degradation in the renal tubules [32]. Normal renal function removes 12% of circulating leptin while in patients with CKD, there is virtually no renal uptake of leptin [32]. If ZAG breakdown occurs as it has been described for leptin in renal tubules, a decrease in GFR and/or tubular reabsorption could explain the rise of plasma ZAG concentration observed in CKD patients. An alternative hypothesis is that ZAG secretion could be specifically increased in uremic subjects. Supporting this idea, we recently demonstrated that uremic plasma stimulates ZAG production by adipocytes *in vitro* and that 5/6 nephrectomized rodents exhibited a significantly increased ZAG protein content in WAT associated with a significant decrease in WAT deposition [33]. We further showed that subcutaneous white adipose tissue biopsies from patients with end-stage renal disease exhibited a higher content of ZAG (5.7 folds) than age-matched controls [33], raising the hypothesis that deregulation of ZAG secretion could also contribute to the increased plasma ZAG concentration reported ESRD patients. Although Ryden et al reported that there was no significant contribution of WAT to the circulating levels of ZAG [34], we can hypothesize that an up-regulation of ZAG production by some tissues could contribute to the increased plasma ZAG concentration. We noticed that plasma ZAG concentration increased during HD independently of hemoconcentration (**Figure 3**) suggesting that additional ZAG is secreted in the organism during HD process. Inflammation, shear stress, oxidative stress, catecholamine secretion or lack of biocompatibility, associated with HD could enhance ZAG secretion. In good agreement, we found a significant increase in plasma ZAG concentration in post-HD patient compared to PD patients (P = 0.004). This result suggests that the increase in circulating ZAG that occurs during HD process is related to factors that are lacking in PD. Glucocorticoids are described to increase ZAG secretion [35], [36]. And cortisol level has been shown to be increased by hemodialysis session [37] suggesting that glucocorticoids could enhance ZAG secretion and contribute to the perdialytic increase in plasma ZAG concentration. In contrast, inflammatory cytokines such as TNF-alpha were shown to decreased ZAG secretion [38]–[40]. The hemodialysis procedure has been associated with enhanced sympathetic activity (recently reviewed in [41]). When the plasma concentration of norepinephrine (NE) was used as a measure of sympathetic outflow, plasma NE was shown to be increased above values in 45% of hemodialysis patients [42]. Beta agonists were described to increase ZAG secretion in white adipose tissue, sympathetic overactivity/catecholamine release could therefore contribute to the perdialytic increase in ZAG. Tedeschi et al (2012) indeed reported a relationship between plasma norepinephrine and ZAG in heart failure patients [43].

The physiological consequences of increased ZAG concentration in renal failure remain to be elucidated. Axelsson et al [28] reported that plasma glycerol concentration (normalized to body fat mass) was increased in CKD stage 5 suggesting an increased lipolytic activity from their white adipose tissue (WAT). In contrast, we failed to show any increase of plasma glycerol in CKD patients (with or without normalization to body fat mass) or any relationship between plasma glycerol and ZAG concentrations. From our data, there is therefore no line of evidence that ZAG accumulation could contribute to PEW in CKD patients as observed in patients with cancer cachexia. ZAG could however contribute to metabolic disturbances since Philipp et al [17] reported that ZAG serum levels negatively correlated with fasting insulin and homeostasis model assessment of insulin resistance (HOMA-IR). Olofsson et al [44] also noticed that ZAG serum levels correlated with serum levels of cholesterol in healthy subjects. Thus, as described in patients with cancer [45], ZAG could contribute to CKD-associated metabolic disturbances, such as insulin resistance and dyslipidemia.

A negative correlation was found between ZAG concentration and plasma albumin (r_s_ = −0.282, p<0.05) and positive correlation between ZAG and urea (r_s_ = 0.283, P<0.01). We however failed to find a significant correlation between plasma albumin level and urea (r_s_ = −0.005, P = 0.953) that does not support an association with wasting. The significant correlation found between plasma ZAG and urea could be explained by the fact that level of urea reflects the accumulation of many uremic toxins that could stimulate ZAG production in CKD [33]. In an animal study, ZAG was further described to exert antiproliferative activity on epithelial tubular cells after acute kidney injury [46] suggesting that accumulation of ZAG could maybe compound renal dysfunction. Further studies are however needed to decipher the pathophysiological role of ZAG in ESRD.

In conclusion, in this study, we report that ZAG protein accumulates in plasma in ESRD and hemodialysis patients but this increase is not directly correlated to the decrease in GFR. In our study, plasma ZAG concentration was not associated with PEW. The main limitation of the present study is however that it is observational and therefore that it is difficult to make causal inference. Further prospective studies are needed to better elucidate the potential role of ZAG in end-stage renal disease.
